# Genetics and genomics of disease resistance in salmonid species

**DOI:** 10.3389/fgene.2014.00415

**Published:** 2014-11-26

**Authors:** José M. Yáñez, Ross D. Houston, Scott Newman

**Affiliations:** ^1^Faculty of Veterinary and Animal Sciences, University of ChileSantiago, Chile; ^2^Aquainnovo, Puerto MonttChile; ^3^The Roslin Institute and Royal (Dick) School of Veterinary Studies, University of EdinburghMidlothian, UK; ^4^Genus plc, HendersonvilleTN, USA

**Keywords:** salmon, disease resistance, breeding programs, QTL, genomic selection

## Abstract

Infectious and parasitic diseases generate large economic losses in salmon farming. A feasible and sustainable alternative to prevent disease outbreaks may be represented by genetic improvement for disease resistance. To include disease resistance into the breeding goal, prior knowledge of the levels of genetic variation for these traits is required. Furthermore, the information from the genetic architecture and molecular factors involved in resistance against diseases may be used to accelerate the genetic progress for these traits. In this regard, marker assisted selection and genomic selection are approaches which incorporate molecular information to increase the accuracy when predicting the genetic merit of selection candidates. In this article we review and discuss key aspects related to disease resistance in salmonid species, from both a genetic and genomic perspective, with emphasis in the applicability of disease resistance traits into breeding programs in salmonids.

## INTRODUCTION

Farming of salmonid species is one of the largest aquaculture industries, with a worldwide production of approximately 1.9 million tons of high value product in 2010 (Food and Agriculture Organization of the United Nations [FAO], 2012). As in other animal production systems, the success and sustainability of salmonid aquaculture largely depends on the control of diseases. A clear example of the negative impact of infectious diseases in salmon farming is the unprecedented economic loss caused by outbreaks of the viral disease infectious salmon anemia (ISA) between 2007 and 2009 in Chile (Asche et al., 2010).

Genetic improvement programs are focused on increasing economic return of aquaculture systems via selective breeding (Gjedrem, 2012). In this regard, all heritable and economically relevant traits should be included in the breeding objective. Thus, in salmonid species, traits such as growth rate, flesh color, and resistance to viral, bacterial, and parasitic diseases should be included (Gjedrem, 2000, 2012). Selective breeding can utilize trait information recorded on selection candidates themselves or, particularly in the case of disease or invasive traits, on relatives. Until now, salmon breeding programs have typically included disease resistance based only on information from relatives, which affects the degree of genetic progress achievable on each generation. This is because of the lower accuracy of estimated breeding values (EBVs) when using only sib information compared to the accuracy obtained when using information of the selection candidates themselves (Falconer and Mackay, 1996).

Recent advances in molecular biology techniques, such as next generation sequencing and high throughput genotyping methods, have helped identify genetic variants influencing phenotypic variation for different traits in a wide range of organisms (Goddard and Hayes, 2009). Molecular markers can be used for a variety of applications in livestock and aquaculture species, such as strain and hybrid identification, genetic variability and genetic diversity evaluation, parentage analyses, quantitative trait loci (QTL) mapping, marker assisted selection (MAS), and genomic selection (GS; Liu and Cordes, 2004; Goddard and Hayes, 2009). Information from a few molecular markers linked to QTL (i.e., genomic regions harboring genes with a significant effect on the trait) might be implemented in breeding schemes through MAS, if they explain a high proportion of genetic variation in the trait. Additionally, the information of 1000s of markers might be simultaneously incorporated into genetic evaluation to estimate genomic breeding values (GEBVs; Meuwissen et al., 2001). These marker-based methods may be particularly useful for the improvement of traits that are complicated or impossible to measure directly on selection candidates, as is the case of resistance to disease (Sonesson and Meuwissen, 2009; Villanueva et al., 2011; Taylor, 2014). A typical first step to implement MAS or GS is to quantify the level of genetic variation in the trait by dissecting its genetic architecture. In salmonids, there is limited information on the genetic architecture of disease resistance. Nevertheless, it is expected that more knowledge on the QTL or genes affecting disease resistance traits will be revealed in the near future, facilitated by the increasing availability of genomic resources and better understanding of the biology of immune response in these species.

This paper reviews aspects of conventional breeding to improve disease resistance in salmonids and the application of molecular tools for the identification of genetic factors involved in these traits. Additionally, the incorporation of molecular information into breeding schemes to improve disease resistance is discussed.

## IMPORTANCE OF DISEASE CONTROL IN SALMON FARMING

The health status of farmed fish is one of the main factors affecting the economic return in the salmon industry. Despite scientific, professional, and technical strategies aimed at improving health management, many novel pathological conditions have emerged in salmonid fish species worldwide in recent decades. A detailed description of each disease affecting culture of salmonid species would greatly exceed the purpose of this review. However, some particular examples are discussed to demonstrate the large economic impact diseases can cause in salmon production.

One of the most striking cases affecting salmon farming was the economic crisis triggered by ISA virus outbreaks since mid-2007 in Chile. The production of Chilean Atlantic salmon suffered a dramatic decrease due to increasingly frequent outbreaks between 2007 and 2009. In fact, total production of Atlantic salmon between 2005 and 2010 decreased by more than 60% in volume (Asche et al., 2010). Currently, ISA virus outbreaks appear to be controlled to a low number of events per year in Europe and North and South America. However, the prevalence and emergence of other viral diseases is still of concern. In Northern European countries including Norway and Scotland, ISA outbreaks have been rare in recent years. Nevertheless, infectious pancreatic necrosis (IPN), caused by an aquatic birnavirus, has caused large levels of mortality in Europe, particularly during the window of susceptibility following transfer from freshwater to seawater (Roberts and Pearson, 2005). Other viral diseases have also emerged and pose serious threats to salmon aquaculture, such as skeletal muscle inflammation (HSMI) – a piscine reovirus – and pancreas disease (PD) – an alphavirus, which has shown an increase in recent years (Biering et al., 2012). These viruses cause direct economic losses through mortality and indirect losses through reduced growth rate and treatment costs.

Among bacterial diseases with a negative impact on salmon farming, salmon rickettsial syndrome (SRS) caused by the Gram-negative bacterium *Piscirickettsia salmonis*, is one of the main sanitary challenges in Chilean salmon industry. This disease affects different salmonid species, including Atlantic salmon (*Salmo salar*), coho salmon (*Oncorhynchus kisutch*), and rainbow trout (*O. mykiss*; Fryer and Hedrick, 2003) and can generate economic losses equivalent to 25% of total profit in salmon exports in Chile (Rozas and Enríquez, 2014). Other bacterial diseases, such as those caused by *Aeromonas salmonicida*, *Vibrio anguillarum*, and *Vibrio/Aliivibrio salminonicida*, are recognized to be efficiently controlled by vaccination and do not currently represent a major economic threat for salmon (Biering et al., 2012).

In terms of parasitic diseases, two different species of sea lice, *Lepeophtheirus salmonis* and *Caligus rogercresseyi*, are most detrimental parasites for salmon farming at a worldwide level. In this regard, it has been estimated that on average, the economic impact of sea lice infestation is about 6% of the total value produced by the world salmon industry (Costello, 2009). An emerging threat to salmon production worldwide is Amoebic Gill Disease (AGD), which has been the major disease of farmed salmonid production in Tasmania for several decades (Mitchell and Rodger, 2011) and has appeared relatively recently in most major salmon-producing countries (Ruane and Jones, 2013). The free-living amoebic protozoan *Neoparamoeba pemaquidensis* is the primary causative agent for the disease that can cause serious morbidity and reduced growth, in addition to increasing susceptibility to other pathogens (Mitchell and Rodger, 2011).

The measures used for prevention and treatment (vaccinations, antibiotics, and antiparasitic drugs, biosecurity measures) of some of the diseases presented above have typically been only partially effective in field conditions (Bravo et al., 2013; Jones et al., 2013; Rozas and Enríquez, 2014). Where effective vaccines do exist, administration typically requires individual handling and treatment of all production fish, which can be expensive and impractical in a large-scale production environment. Due to the fact that improvement in economic efficiency of salmon farming is dependent on disease prevention and control, (Asche and Roll, 2013) it is imperative to develop alternative effective and sustainable strategies. Genetic improvement of disease resistance represents a feasible solution to increase the sanitary status in animal production (Stear et al., 2001; Bishop, 2010). In this regard, there is increasing scientific literature aiming at both quantifying levels of host genetic variation for resistance against different diseases and identifying the specific genetic factors that influence these traits in salmonid species, as discussed below.

## CONVENTIONAL BREEDING FOR DISEASE RESISTANCE IN SALMONIDS

Resistance to diseases can be defined as the ability of the host to limit infection by reducing pathogen replication (Råberg et al., 2007; Doeschl-Wilson et al., 2012). Selecting animals with increased resistance to specific diseases is a feasible method to improve productivity and animal welfare and offers advantages over other control methods against infection, such as the cumulative and permanent benefits of the improved resistance (Stear et al., 2001; Bishop, 2010). Disease resistance has been a target trait for the salmon breeding industry for at least 20 years, with a Norwegian salmon breeding program including resistance to bacterial and viral diseases into its breeding goal since 1993 (Gjøen and Bentsen, 1997). However, the study of disease resistance and its incorporation into breeding programs can be hindered by the difficulty in determining and measuring accurate and appropriate phenotypes (Bishop and Woolliams, 2014). This in turn influences the accuracy of disease resistance EBVs that can be achieved. Another limiting step is that disease information is typically only available from relatives of the selection candidates and not directly from the candidates themselves. In the following, we review the main aspects of breeding for resistance to infectious diseases in salmonids and discuss current status and future directions of research in this area.

### CHALLENGE AGAINST PATHOGENS

Host resistance to viral and bacterial pathogens can often be measured, in practical terms, as survival (and/or mortality) of individuals during an outbreak (Ødegård et al., 2011). Data and samples from field outbreaks can be used opportunistically to make inference about genetic resistance to infectious diseases. For this purpose, it is necessary that the pedigree of the population be accurately determined, often using genetic markers or electronic tagging of the fish (Guy et al., 2006). However, using the information from field outbreaks has some disadvantages, such as difficulty to identify the exact cause of death because the factors that influence survival under these conditions are likely to be diverse. Furthermore, the availability of information depends on the occurrence of high-mortality outbreaks, which are usually prevented or controlled to avoid serious economic loss. Moreover, the inference of pedigree using molecular markers can be expensive and laborious. Therefore, survival data are often obtained from experimental challenges, which can readily be standardized to control other variables and potentially allow a clearer interpretation of the results. In this case, it is necessary that a high genetic correlation between the trait measured in experimental and field conditions exists. High genetic correlations (*r*_g_ ≥ 0.95) between field trials and experimental challenges to furunculosis in Atlantic salmon have been reported (Gjøen et al., 1997; Ødegård et al., 2006), suggesting that results from experimental challenges are likely to be directly applicable to commercial production systems. Therefore, challenge tests will often be more accurate and reliable than field outbreaks, due to decreased environmental variability and higher practical feasibility. In fact, challenge testing is currently used to select for resistance to viral, bacterial, and parasitic diseases in breeding programs for Atlantic salmon and rainbow trout (Gjøen and Bentsen, 1997; Leeds et al., 2010; Yáñez and Martínez, 2010; Ødegård et al., 2011; Gjedrem, 2012; Wiens et al., 2013a).

### IMMUNOLOGICAL AND PHYSIOLOGICAL VARIABLES AS INDIRECT MEASURES OF RESISTANCE

Direct genetic selection for improved disease resistance based on challenge testing can be costly and time consuming, and has negative animal welfare implications. Furthermore, selection decisions using this strategy can only be carried out using information from relatives and not the candidates themselves. Indirect selection based on the measurement of other characteristics that are genetically correlated with disease resistance, would simplify the data collection and allow the incorporation of individual information. Some studies have aimed at determining the genetic variation of physiological and immunological variables, and the correlation between them and survival in challenge tests in salmon. Examples of variables that have been studied to date are hemolytic activity of serum and lysozyme activity (Røed et al., 1993; Lund et al., 1995), plasma levels of cortisol (Fevolden et al., 1993; Weber et al., 2008), and levels of IgM and antibody titer (Lund et al., 1995), serum α2-antiplasmin (Salte et al., 1993), bactericidal and complement activity (Hollebecq et al., 1995). However, even when some studies show significant correlations between resistance and immune parameters, the proportion of the total variation in survival that could be explained by immune variables has been considered too low to be useful as a selection criterion. Hence, the prediction of breeding values for survival based on these variables may not be practically useful (Gjøen and Bentsen, 1997). This may be due in part to the complexity of the mechanisms involved in the immune response and the large number of factors that may be involved in disease resistance, which results in a great difficulty when trying to use the information from a single parameter for the genetic evaluation of disease resistance.

### GENETIC VARIATION IN RESISTANCE TO INFECTIOUS DISEASES

A requirement to improve a trait by means of artificial selection is that sufficient genetic variation for this trait exists in the population. Heritability is the proportion of the total phenotypic variance that is attributable to additive genetic variation (Falconer and Mackay, 1996). For disease resistance traits, heritability estimates can vary due to differences in trait definitions and the statistical models used in the analysis (Yáñez and Martínez, 2010; Ødegård et al., 2011). For example, some studies consider the binary trait of survival or mortality as a measure of resistance, whereas others consider the survival time following challenge. These require different statistical approaches to analysis, and are likely to inform on different components of the host response to infection. Nonetheless, there have been several studies aimed at determining levels of additive genetic variation for resistance to different diseases affecting salmonid species (see **Table 1**). The results from these studies show the feasibility of improving disease resistance through genetic improvement and the potential of this approach for helping in the control of disease problems in salmonids.

**Table 1:** Heritabilty values (*h*^***2***^) and their SE for resistance to different infectious and parasitic diseases in salmonid species.

Salmonid species	Disease agent	*h*^*2*^ ± SE	Reference
*Salmo salar*	*Neoparamoeba spp.*	0.40 ± 0.08 – 0.49 ± 0.09	Taylor et al. (2009)
	*Renibacterium salmoninarum*	0.2 ± 0.1	Gjedrem and Gjøen (1995)
	*Aeromonas salmonicida*	0.48 ± 0.17	Gjedrem et al. (1991)
	*A. salmonicida*	0.34 ± 0.13	Gjøen et al. (1997)
	*A. salmonicida*	0.38 ± 0.09	Ødegård et al. (2006)
	*A. salmonicida*	0.43 ± 0.02	Ødegård et al. (2007b)
	*A. salmonicida*	0.59 ± 0.06	Olesen et al. (2007)
	*A. salmonicida*	0.62	Kjøglum et al. (2008)
	*A. salmonicida*	0.47 ± 0.05	Gjerde et al. (2009)
	*Gyrodactylus salaris*	0.32 ± 0.10	Salte et al. (2009)
	IPNV	0.43	Guy et al. (2006)
	IPNV	0.5	Wetten et al. (2007)
	IPNV	0.55	Kjøglum et al. (2008)
	ISAV	0.13 ± 0.03	Gjøen et al. (1997)
	ISAV	0.16 ± 0.01	Ødegård et al. (2007a)
	ISAV	0.32 ± 0.02	Ødegård et al. (2007b)
	ISAV	0.24 ± 0.03	Olesen et al. (2007)
	ISAV	0.37	Kjøglum et al. (2008)
	ISAV	0.40 ± 0.04	Gjerde et al. (2009)
	*Caligus elongatus*	0.22	Mustafa and MacKinnon (1999)
	*Lepeophtheirus salmonis*	0.14 ± 0.02	Kolstad et al. (2005)
	*Lepeophtheirus salmonis*	0.07 ± 0.02	Glover et al. (2005)
	*Vibrio anguillarum*	0.38 ± 1.07	Gjøen et al. (1997)
	*V. salmonicida*	0.13 ± 0.08	Gjedrem and Gjøen (1995)
	*Caligus royercresseyi*	0.10 ± 0.03	Yáñez et al. (2014a)
	*Caligus royercresseyi*	0.12 ± 0.07 – 0.34 ± 0.07	Lhorente et al. (2012)
	SPDV	0.21 ± 0.005	Norris et al. (2008)
	*Piscirickettsia salmonis*	0.11 ± 0.02 - 0.41	Yáñez et al. (2013)
	*P. salmonis*	0.18 ± 0.03	Yáñez et al. (2014a)
*Salvelinus fontinalis*	*Aeromonas salmonicida*	0.51 ± 0.03	Perry et al. (2004)
*Oncorhynchus tshawytscha*	*R. salmoninarum*	0 ± 0.05	Beacham and Evelyn (1992)
	*A. salmonicida*	0.21 ± 0.14	Beacham and Evelyn (1992)
	*V. anguillarum*	0.14 ± 0.11	Beacham and Evelyn (1992)
*O. kisutch*	*R. salmoninarum*	0.53 ± 0.16	Withler and Evelyn (1990)
*O. mykiss*	*Yersinia ruckeri*	0.21 ± 0.05	Henryon et al. (2005)
	*Flavobacterium psychrophilum*	0.35 ± 0.09	Silverstein et al. (2009)
	*F. psychrophilum*	0.23 ± 0.03	Leeds et al. (2010)
	*F. psychrophilum*	0.53 ± 0.09	Vallejo et al. (2010)
	*F. psychrophilum*	0.07 ± 0.02	Henryon et al. (2005)
	VHS	0.63 ± 0.26	Dorson et al. (1995)
	VHS	0.11 ± 0.1	Henryon et al. (2005)

### GENETIC CORRELATIONS BETWEEN DISEASE RESISTANCE AND OTHER TRAITS

The potential to simultaneously improve resistance to different diseases and other economically important traits is partly dependent on the genetic correlations between the traits. Few studies to date have aimed to determine the genetic correlations for disease resistance traits in salmon. Some studies in Atlantic salmon have indicated positive genetic correlations between resistance to bacterial diseases such as furunculosis, BKD, and cold water Vibriosis (Gjedrem and Gjøen, 1995; Gjøen et al., 1997). Weak negative genetic correlations between resistance against ISA and bacterial diseases such as furunculosis, vibriosis, and cold-water vibriosis have been reported (Gjøen et al., 1997). However, positive genetic correlations between resistance against ISA virus and furunculosis have also been reported (Ødegård et al., 2007b). In rainbow trout, weak genetic correlations between viral hemorrhagic septicemia (VHS) and bacterial diseases such as enteric redmouth disease (ERM) and rainbow trout fry syndrome have been found (Henryon et al., 2005). Kjøglum et al. (2008) reported only weak genetic correlations when they estimated genetic correlations between resistance to IPN, ISA, and furunculosis. Verrier et al. (2013b) also failed to detect any genetic correlation between host resistance to two rhabdoviral pathogens (VHS and Infectious Hematopoietic Virus). Additionally, weak genetic correlations have also been calculated between resistance against SRS and *C. rogercresseyi* in Atlantic salmon (Yáñez et al., 2014a). In general, these results suggest no clear-cut relationship between genetic resistance to one pathogen and genetic resistance to another pathogen.

Moreover, it is important to know the genetic correlations between disease resistance and other economically important traits in salmon production, especially for selection index development. Previous reports of correlations between disease resistance and production traits have ranged from zero (Silverstein et al., 2009), low to moderately negative (Henryon et al., 2002), inconsistent (Beacham and Evelyn, 1992; Henryon et al., 2002), and low to moderately positive (Gjedrem et al., 1991; Perry et al., 2004; Yáñez et al., 2014a). Thus, it is important to evaluate these parameters for each particular breeding population to maximize the impact of selecting for improved disease resistance. Additionally, the relationship between ploidy levels and disease resistance is of interest because all female triploid rainbow trout are advantageous for production. Weber et al. (2013) showed that triploid fish are generally slightly more susceptible to Bacterial Coldwater Disease than diploids, but that selection for improved resistance in diploids is also effective for triploid production.

### RESISTANCE VERSUS TOLERANCE

If resistance is defined as an individual’s ability to block the reproduction of a pathogen, then disease tolerance can be defined as the ability to limit the impact of infection on a host (Råberg et al., 2007; Doeschl-Wilson et al., 2012). Information relating to the genetic basis of disease tolerance has been sparse in animal genetics studies to date (Doeschl-Wilson et al., 2012). However, it is important to disentangle resistance from tolerance because the appropriate trait to select for may differ depending on the disease, host species, and environment. It is also possible that the two traits will be antagonistic, which could result in inadvertent undesirable outcomes of selection for disease resistance (Doeschl-Wilson et al., 2012). The implications of selection for resistance or tolerance on the host–pathogen interaction and pathogen evolution have also been considered. For example, selection for resistance *per se* may lead to selection pressure for higher virulence in the pathogen, whereas selection for tolerance could result in co-existence of pathogen and host with minimal impact on the performance of the host population. Therefore, in future disease studies and in selective breeding programs, it will be important to carefully consider the optimal disease trait to target for maximum benefit at the population level, but also the analytic issues of resistance versus tolerance.

## STRATEGIES FOR GENETIC DISSECTION OF DISEASE RESISTANCE TRAITS

Major advances in nucleotide sequencing, ever-improving bioinformatics pipelines, and high-throughput genotyping tools have helped to identify genes associated with complex traits in various species of vertebrates. In salmonids, this is reflected in the substantial increase in genomic resources for these species during recent years and, for example, in the formation of an international collaboration to sequence the Atlantic salmon genome (Davidson et al., 2010). The salmon genome has undergone a relatively recent duplication and has a very high content of long repeat elements. This has hampered the sequencing and assembly of a reference genome for salmonids. However, a high-quality reference sequence has been published for rainbow trout (Berthelot et al., 2014) and is available for Atlantic salmon (Davidson et al., 2010). Further, high density SNP genotyping arrays were recently developed for Atlantic salmon (Houston et al., 2014; Yáñez et al., 2014b), and a lower density platform (Lien et al., 2011) has previously been used for QTL mapping and population genetics. Currently, these genomic resources are increasingly being used for the identification of the genetic factors involved in the resistance to different diseases in salmonids. The strategies used in the study of the genetic architecture of disease resistance can be classified as: (i) candidate gene approaches, (ii) QTL mapping, and (iii) gene expression studies.

### CANDIDATE GENE APPROACH

Candidate gene theory states that a significant proportion of phenotypic variance of one trait in a population is determined by the presence of polymorphisms within genes known to be involved in the physiological regulation of that trait (Rothschild and Soller, 1997). This approach requires previous knowledge on the biology of the species, biochemical pathways, and especially gene sequences, to study the variation within specific candidate genes. In aquaculture species, the availability of annotated gene sequences of known function is typically low, but is likely to increase in the short term with the use of high-throughput sequencing and ongoing genome sequencing projects.

In vertebrates, the major histocompatibility complex (MHC) has attracted much attention in studies of association between genetic variants and disease resistance. However, other genes are likely to play an important role in the mechanisms of disease resistance in production animals, model organisms, and humans (Hill, 1999; Qureshi et al., 1999). To our knowledge, there are no studies aimed at establishing association between candidate genes, other than the MHC, and resistance to infectious diseases in salmonid species.

#### Major histocompatibility complex

The MHC is a multigene family that acts at the interface between the immune system and infectious pathogens. The MHC gene family comprises two subfamilies: class I and II. Both classes are membrane glycoproteins involved in the processing and removal of pathogens (Thorgaard et al., 2002). MHC genes have been identified, cloned, and characterized in Atlantic salmon, rainbow trout, and other salmonids (Grimholt et al., 1993; Hordvik et al., 1993; Hansen et al., 1996; Shum et al., 1999, 2002). Furthermore, it has been shown that these genes are highly polymorphic in these species (Grimholt et al., 1994, 2002; Miller and Withler, 1996; Hansen et al., 1999; Garrigan and Hedrick, 2001; Aoyagi et al., 2002). As in other vertebrates there are two types of class I genes; the UAA which are highly divergent, non-polymorphic and expressed at low levels, and the UBA which are polymorphic, expressed at high levels in spleen, and with structural features similar to those of class Ia molecules (classical) which present antigen to T lymphocytes (Shum et al., 1999). The class II genes are divided into Class II A (DAA) and II B (DAB), depending on whether encoding α or β chain of the molecule, respectively (Grimholt et al., 2000; Stet et al., 2002). Both loci (DAA and DAB) co-segregate as haplotypes, suggesting a close physical linkage between them in Atlantic salmon (Stet et al., 2002).

The association between a polymorphism linked to MHC class II genes and resistance to virus infectious hematopoietic necrosis (IHN) in backcrosses of rainbow trout and cutthroat trout (*O. clarki*) has been found, but was relatively weak and dependent on the family analyzed (Palti et al., 2001). Suggestive associations between rainbow trout MHC class IB alleles and bacterial cold water disease have also been shown (Johnson et al., 2008). In Atlantic salmon, the association between MH class IIB alleles and resistance against *A. salmonicida* has been reported (Langefors et al., 2001; Lohm et al., 2002). In the same species, MHC class I and class II variants have been associated with susceptibility to IHN (Miller et al., 2004) and resistance to furunculosis and ISA (Grimholt et al., 2003; Kjøglum et al., 2006). Although associations between MH gene variants and disease resistance have been established, the MHC is most likely not the only factor influencing genetic variation in disease resistance. For instance, in Atlantic salmon, a non-MHC effect for resistance to IPN, furunculosis and ISA has been detected (Kjøglum et al., 2005). Because disease resistance traits will typically be polygenic in nature, it is important to consider variants in a genome-wide context and possible interactions that can occur between genes (epistasis), which hinders the candidate gene approach as a comprehensive strategy for incorporating molecular information into the genetic evaluation of these traits.

### QTL MAPPING

Quantitative trait loci mapping is a strategy providing information on the location and effect of the gene variants influencing complex quantitative traits, but without prior hypotheses. The QTL detection methodologies are based on the use of (typically anonymous) DNA markers dispersed throughout the genome to identify genomic regions involved in the genetic variation of a particular trait, by means of statistical analyses utilizing the co-segregation between markers and the (unknown) causative variants.

#### DNA markers

The development of DNA markers has had a major impact on studies of genetic variation in animals and fish. The categories of DNA markers widely used historically include Restriction Fragment Length Polymorphisms (RFLP), Random Amplified Polymorphic DNA (RAPD), Amplified Fragment Length Polymorphism (AFLP), microsatellites, and single nucleotide polymorphisms (SNPs). More recently, SNP markers have become the predominant marker due to their abundance, ease of discovery and low cost of genotyping per locus (Houston et al., 2014). However, each of these marker types vary from each other in their mode of inheritance (i.e., dominant or codominant), identification and detection methods, number of spanning loci and polymorphic information content (Liu and Cordes, 2004).

Microsatellites are tandem repeat nucleotide sequences that generally span between one to six base pairs. Different alleles are generated due to the variation in the number of repeats. Their main useful features for genetic studies include high variability, codominant inheritance, abundance, and wide distribution across the genome. One of the major advantages of microsatellites is their high degree of polymorphism, with 10s of alleles often observed at a single locus in an outbred population. Additionally, their genotyping is typically based on a simple DNA amplification by means of polymerase chain reaction (PCR). Thus, genotyping can be relatively rapid, cheap, and the amount of DNA required is minimal (nanograms). However, the scoring of microsatellites often requires optimization and manual input, which reduces their scalability for large genetic studies. There are a considerable number of microsatellites identified for different salmonid species, available for use in genetic studies (e.g., Cairney et al., 2000; Gilbey et al., 2004; Phillips et al., 2009).

Historically, AFLPs (which typically score SNPs) had the advantage of being generated more easily and cheaply than SNPs and microsatellites, because previous knowledge on genomic sequence is not needed for their generation. However, their mode of inheritance, similar to RAPDs, is dominant, i.e., it is not possible to distinguish between heterozygous and one category of homozygous genotypes without the use of special equipment and software (Piepho and Koch, 2000). This reduces the amount of information provided by these kinds of markers.

Single nucleotide polymorphismss have several key advantages for genetic studies, which have led to a rapid increase in their popularity over recent years. Some of these include high abundance, amenability to automated scoring in large numbers, simultaneous assays of thousands of markers (e.g., using SNP arrays) and presence in both coding and non-coding regions. Therefore, SNPs have been applied recently for construction of dense genetic maps, which can be used for fine mapping of QTLs and facilitate the identification of causative genes involved in the genetic variation of specific characters (e.g., Lien et al., 2011; Gonen et al., 2014). Recently, there has been a rapid increase in available SNPs for salmonid species, mainly for Atlantic salmon and rainbow trout (Hayes et al., 2007; Sanchez et al., 2009; Everett et al., 2011, 2012; Hohenlohe et al., 2011; Lien et al., 2011; Houston et al., 2012, 2014; Salem et al., 2012). Additionally, SNP arrays are available for simultaneous genotyping of 10s to 100s of 1000s of markers in rainbow trout (Palti et al., 2014b) and Atlantic salmon (Houston et al., 2014; Yáñez et al., 2014b). These SNP resources are likely to be increasingly applied to high-resolution mapping of disease resistance genes in salmonid species.

In recent years, the same sequencing technology that has led to the increased ease of high-density SNP discovery described above has enabled direct genotyping of individual fish based on sequence information alone. Such techniques are collectively termed ‘genotyping by sequencing’ (GBS). Although GBS techniques encompass a diverse range of laboratory and bioinformatic pipelines, they are all based on the principle of using nucleotide barcodes ligated to the fragmented genomic DNA of individual fish and high-throughput sequencing in multiplexed pools (Davey et al., 2013). Partly due to the lack of established genomics resources for many farmed fish species, the aquaculture genetics community has been early adopters and developers of these techniques. In particular, RAD sequencing has been utilized in both Atlantic salmon (e.g., Houston et al., 2012) and rainbow trout (Palti et al., 2014a) as a conduit to incorporating genomic information into aquaculture breeding programs. While the application of GBS has huge potential for applied research in salmonids, there are also some challenges in managing and interpreting these datasets. For example, discovery of ‘false’ SNPs using sequencing can occur, particularly with the recent whole genome duplication of the salmonid species. Therefore, species-tailored bioinformatics pipelines are typically required to minimize these issues.

#### Detection of QTL affecting disease resistance in salmonid species

Salmon may be more likely than terrestrial farmed species to have QTL of major effect because they have had fewer generations of selection in the farmed environment and therefore the standing genetic variation for traits of economic importance may still be very large. The development of a genetic map based on linkage between genetic markers is the first step towards the identification of QTL. To date, several linkage maps have been constructed using different marker types for rainbow trout (e.g., Young et al., 1998; Sakamoto et al., 2000; Nichols et al., 2003; Guyomard et al., 2006; Rexroad et al., 2008; Palti et al., 2011, 2012; Guyomard et al., 2012); Atlantic salmon (Gilbey et al., 2004; Moen et al., 2004b; Lien et al., 2011; Gonen et al., 2014); coho salmon (McClelland and Naish, 2008); brown trout (*Salmo trutta*; Gharbi et al., 2006); Arctic Trout (*Salvelinus alpinus*; Woram et al., 2004); and sockeye salmon (*Salmo nerka*; Everett et al., 2012).

Using microsatellite markers, two QTL with major effects on resistance to IPN in rainbow trout has been detected. These loci explained a large proportion (27 and 34%) of the phenotypic variation in a family from a backcross between a strain susceptible to IPN (YK-RT101) and a resistant one (YN-RT201; Ozaki et al., 2001). Using AFLP and microsatellite markers, QTLs for IHN resistance have been identified in three different linkage groups of the same species (Rodriguez et al., 2004). For the same disease, RFLP markers were associated with resistant and susceptible families in backcrosses of rainbow trout and cutthroat trout (Palti et al., 1999). Extensive research into the genetic architecture of resistance to Bacterial Coldwater Disease has also been undertaken, with evidence for QTL of major effect (Wiens et al., 2013b; Vallejo et al., 2014a,b). Additionally, major QTL have been detected for resistance to whirling disease caused by the myxosporean parasite *Myxobolus cerebralis* (Baerwald et al., 2010) and VHS (Verrier et al., 2013a).

In Atlantic salmon, using AFLP markers, two QTL associated with resistance to ISA have been detected in two full sib families (Moen et al., 2004a). One of these QTLs has been validated using microsatellite markers from a higher number of genotyped fish. This QTL explained 6% of the phenotypic variation for resistance to ISA and has been mapped to linkage group VIII of the Atlantic salmon genome (following SALMAP notation; Moen et al., 2007). In the same species, a major QTL for resistance to IPN has been identified using data collected from a ‘field’ seawater outbreak of the disease (Houston et al., 2008a). The QTL detection strategy involved utilizing the low male recombination rate observed in salmonids by using just two to three microsatellites per chromosome and male segregation to determine linkage groups with a significant effect, and secondly, a higher number of markers per linkage group. Female segregation was used to confirm the previously detected QTL and position it within the linkage group (Houston et al., 2008a). The major QTL, mapped to linkage group 21, was subsequently confirmed by analyzing nine additional families and a higher saturation of markers (Houston et al., 2008b). The same QTL was then confirmed and fine mapped in an independent population, and haplotypes of markers that could predict the genotype at the QTL were identified based on the linked microsatellites (Moen et al., 2009). Using a restriction-site associated DNA sequencing approach (RAD sequencing), several additional SNP markers linked to the QTL were identified and two SNP markers showed a significant population-level association with resistance in two year classes from an Atlantic salmon breeding population (Houston et al., 2012). The high proportion of the total variance for IPN resistance that this QTL explains has allowed the incorporation of markers linked to it into MAS schemes for the genetic improvement of this trait in Atlantic salmon in both Norway (Moen et al., 2009) and Scotland (Houston et al., 2010).

In general, confidence intervals for mapped QTLs mapped are large. This issue has two consequences: first, widespread confidence intervals might contain a large number of genes (1000s) and, therefore, identification of the causative polymorphism is challenging. Second, the use of markers linked to QTLs in MAS programs is complicated, since the linkage phase between the marker and the QTL throughout the population may be different from family to family. An alternative for QTL fine mapping is to use information from linkage analysis in conjunction with information from linkage disequilibrium (LD) across the population (Meuwissen et al., 2002). Through simulation studies, power and accuracy of this combined approach has been successfully tested for QTL fine mapping, accounting for the structure of commercial salmon populations (Hayes et al., 2006). Additionally, the availability of both high-density SNPs marker panels and high-resolution genetic maps will contribute to detect association between markers and QTLs with higher precision by means of using across-population LD mapping (Goddard and Hayes, 2009). These strategies along with a reference sequence and a consolidated physical map of the genome, will facilitate the identification of causative mutations affecting disease resistance traits through positional studies in salmonid species.

### GLOBAL GENE EXPRESSION

Functional genomics, defined as the application of experimental methods of genomic or systemic coverage to assess gene function using data from structural genomics (mapping and sequencing), has been recognized as an area of primary interest in disease studies (Hiendleder et al., 2005). These methodologies broaden the spectrum of biological research to study, simultaneously, the expression of thousands of genes at the transcriptional level. Currently, genomic resources and new sequencing technologies have helped to assess differential gene expression levels in the response against diseases in salmonid species. These data can help to pinpoint functional genetic variation underlying disease resistance.

An early example, using suppression subtractive hybridization (SSH) and liver samples from individuals injected with a *V. Anguillarum* bacterium and normal individuals, more than 25 genes important in the immune response in rainbow trout were identified, including sequences of proteins of acute phase of inflammation, complement, and coagulation system (Bayne et al., 2001). Using the same technique, genes involved in signal transduction and innate immunity (among others) have been identified as relevant factors in response to a challenge against *A. salmonicida* in Atlantic salmon (Tsoi et al., 2004).

The availability of ESTs (expression sequence tags) and cDNA libraries have allowed the development of DNA microarrays which can be used to study the differential expression patterns of a large number of genes simultaneously in salmonids (Rise et al., 2004b; Ewart et al., 2005; von Schalburg et al., 2005). Using a microarray of human cDNAs, differentially expressed transcripts against a challenge with *A. Salmonicida* have been identified in Atlantic salmon. However, due to species divergence, only 6% of the sequences of the microarray showed detectable hybridization against salmon liver cDNA (Tsoi et al., 2003). Rise et al. (2004a) conducted the first study using a microarray constructed from salmon cDNA libraries. Following this, differential gene expression has been assessed in between macrophages infected and uninfected with *P. salmonis* and hematopoietic kidney from Atlantic salmon individuals challenged or not against the same pathogen. Differentially expressed genes were proposed to be relevant in immune response and as potential biomarkers of infection with *P. salmonis* (Rise et al., 2004a). Additionally, using an Atlantic salmon cDNA microarray including more than 4,000 genes extracted from liver, spleen and hematopoietic kidney, several differentially expressed genes in response to infection by *A. salmonicida* have been found (Ewart et al., 2005). Furthermore, the transcriptomic response against vaccination with an *A. salmonicida* bacterium has been assessed in Atlantic salmon, revealing temporal and tissue differences in terms of expression levels, which may be relevant in establishing protection (Martin et al., 2006). In addition, gene expression profiles in response to a DNA vaccine for the IHN virus has been studied in rainbow trout, identifying 910 genes modulated in the injection site, and also determining the over-expression of genes of the type I Interferon system (IFN-1) in other tissues, suggesting that this system forms the basis of early antiviral immunity (Purcell et al., 2006). In the studies presented above, certain transcripts that showed variation in their expression levels during infection have low levels of homology with well-characterized genes available in public databases and, thus, do not have a known function (Rise et al., 2004a; Ewart et al., 2005; Martin et al., 2006; Purcell et al., 2006). Therefore, studies based on sequences of the encoded proteins aimed at decrypting their role in the immune response against infection are still needed.

To date, few studies have focused on the analysis of differential expression between resistant and susceptible fish for a particular disease. For example, Sutherland et al. (2014) analyzed gene expression profile differences between chum and pink salmon during infections with sea lice (*L. salmonis*) to gain insight into the functional mechanisms underlying the divergent resistance to lice observed in these two species. Zhang et al. (2011) took a similar approach to examine the differences between resistant and susceptible salmon families to the pathogen *A. salmonicida* to highlight the importance of several innate immune response genes. Cofre et al. (2014) also examined differential expression of several candidate genes in families with divergent resistance to IPNV. Finally, Langevin et al. (2012) compared the transcriptional response of resistant and susceptible clonal lines for Bacterial Coldwater Disease. The information provided by this type of analysis might be useful in the discovery of new sets of genes, with or without an assigned function, which may be associated with disease resistance (Walsh and Henderson, 2004). Another possibility is to consider the differential expression levels as a quantitative trait. This may allow identification of QTLs associated with differences in gene expression patterns between resistant and susceptible individuals (eQTL; Pomp et al., 2004; de Koning et al., 2005). However, it remains unclear how the information given by expression analysis can be used in breeding programs (Walsh and Henderson, 2004).

## MOLECULAR MARKER-ASSISTED SELECTION (MAS) AND GENOMIC SELECTION (GS)

While selective breeding based on performance information from the selection candidate and its relatives is a highly successful means of genetic improvement in the trait of interest, utilizing genetic markers can provide an improvement in both genetic gain and selection accuracy (Goddard and Hayes, 2009). Historically, the use of genetic markers required mapping of QTL and therefore a key parameter for subsequent application in MAS programs is the level of LD between markers and causative mutations at a population level (Goddard and Meuwissen, 2005).

One factor reducing the level of LD each generation is recombination. When the LD between the QTL and the marker only exists within families and not across families, recombination can break the association between marker alleles and the QTL between families. Therefore, the linkage phase between the marker and the QTL should be determined in each generation and separately for each family if it is to be utilized in selection (Wientjes et al., 2013). To determine if the marker and QTL are in LD within each family phenotypic records and genotypes are needed on each generation. This makes unattractive the implementation of MAS exploiting only within-family LD (i.e., linkage) between the QTL and markers (Dekkers and Van der Werf, 2007). In the case of disease resistance traits, this means that all families comprising the breeding nucleus would need to be challenged each generation.

Marker assisted selection schemes are likely to be optimal when the markers explain a large proportion of the total variance of the trait, as in the case of the QTL for resistance to IPN in Atlantic salmon (Houston et al., 2008a, 2012; Moen et al., 2009). The information from markers linked to this QTL is being applied in MAS programs in different breeding populations of Atlantic salmon. Although SNP variants associated with IPN resistance in two different year classes of a breeding nucleus have been discovered (Houston et al., 2012), there is still a need to validate if these alleles are associated with resistance in independent Atlantic salmon populations. This will depend on the LD relationship between the markers and the causative mutation in the population(s) of interest. As such, testing the association between markers and IPNV mortality in these populations should be considered a prerequisite for effective commercial application.

Another alternative to exploit the LD at the population level is using information from high density panels of markers to predict GEBVs of selection candidates (Meuwissen et al., 2001). This approach effectively takes into account all markers when estimating the breeding value of the candidate, without the need to surpass a significance threshold for association with a particular trait of interest. The effectiveness of this strategy will also depend on the magnitude of the effects associated with the markers. When the effects of the markers across the entire genome are estimated, these effects can be used to select individuals lacking phenotypes (Meuwissen et al., 2001). As such, in the case of disease resistance, there would be fewer requirements for challenge testing of siblings for diseases of economic importance. Recombination results in decay of LD on each generation and the magnitude of this reduction depend on several population features (Porto-Neto et al., 2014). This means that in practice, it is necessary to corroborate the accuracy in estimating the GEBVs and response selection in each generation.

*Best linear unbiased prediction* (BLUP) combines information from pedigree and phenotypes to predict breeding values (EBVs) of individuals. Molecular markers provide a new source of information, which will give higher accuracy when predicting EBVs. Thus, selection response will be potentially higher in traits in which the accuracy is low, i.e., traits with low heritability or traits that cannot be measured in the selection candidate, such as disease resistance (Sonesson and Meuwissen, 2009; Villanueva et al., 2011; Taylor, 2014). The relative increase in accuracy depends on the amount of variation explained by the markers. It is also possible to combine breeding values for disease resistance and other performance traits into a selection index with specified weightings based on their economic importance for multi-trait genetic improvement.

In livestock species the effects of QTL have been shown to exhibit a moderate leptokurtic gamma distribution, suggesting a small number of loci of large effect and high number of loci of small effect (Hayes and Goddard, 2001), which is likely to be expected in aquaculture species for most economically relevant traits. Therefore, it is expected that more than one marker is necessary to efficiently assist breeding programs using molecular information. The availability of high throughput SNP genotyping platforms will allow the use of these markers in selective breeding for aquaculture species by means of the implementation of GS schemes (Goddard and Hayes, 2009; Sonesson and Meuwissen, 2009; Taylor, 2014). There are currently at least three independent initiatives to generate high density SNP arrays for Atlantic salmon, run by groups from three of the leading producers of this species: Chile, Scotland and Norway. The first of these to be published is that of Houston et al. (2014) whereby approximately 132 K SNP markers were identified and verified in several populations of farmed and wild Atlantic salmon. The Chilean group validated almost 160 K SNP markers from a 200 K SNP platform for Atlantic salmon, genotyping fish representing wild and farmed populations from Europe, North America, and Chile (Yáñez et al., 2014b). Additionally, a 57 K SNP chip is available for rainbow trout (Palti et al., 2014b). Therefore, it can be expected that programs using a high number of markers in population LD will be implemented in the near future. However, it is necessary to determine the economic benefit of using high-density panels, versus the use of low-density panels supported by imputation methodologies and establish a strategy for GS taking into account technical and economic feasibility.

## CONCLUSION

There is increasing information related to the determination of the genetic basis for disease resistance in salmonid species. The rapid development of genomic resources in these species will provide new tools for genetic dissection of these traits. The use of these tools will be of great help in identifying loci involved in the genetic variation of disease resistance. This information will increase our understanding of the underlying biology of resistance to disease. Further, it will be crucial for the implementation of MAS or GS programs that include disease resistance within the breeding objective. These methods will increase the accuracy of selection candidates, thereby improving the selection response. However, the economic feasibility and profitability of the implementation of these new strategies and its comparison with conventional selection schemes must be studied for each particular breeding program in salmonids. It will also be necessary to assess the long-term impact of these strategies on the control of each specific disease, in an epidemiological context.

## Conflict of Interest Statement

The authors declare that the research was conducted in the absence of any commercial or financial relationships that could be construed as a potential conflict of interest.
